# Combined array-comparative genomic hybridization and single-nucleotide polymorphism-loss of heterozygosity analysis reveals complex genetic alterations in cervical cancer

**DOI:** 10.1186/1471-2164-8-53

**Published:** 2007-02-20

**Authors:** Judith N Kloth, Jan Oosting, Tom van Wezel, Karoly Szuhai, Jeroen Knijnenburg, Arko Gorter, Gemma G Kenter, Gert Jan Fleuren, Ekaterina S Jordanova

**Affiliations:** 1 Department of Pathology, Leiden University Medical Center, Leiden, The Netherlands; 2 Department of Molecular Cell Biology, Leiden University Medical Center, Leiden, The Netherlands; 3 Department of Gynecology, Leiden University Medical Center, Leiden, The Netherlands

## Abstract

**Background:**

Cervical carcinoma develops as a result of multiple genetic alterations. Different studies investigated genomic alterations in cervical cancer mainly by means of metaphase comparative genomic hybridization (mCGH) and microsatellite marker analysis for the detection of loss of heterozygosity (LOH). Currently, high throughput methods such as array comparative genomic hybridization (array CGH), single nucleotide polymorphism array (SNP array) and gene expression arrays are available to study genome-wide alterations. Integration of these 3 platforms allows detection of genomic alterations at high resolution and investigation of an association between copy number changes and expression.

**Results:**

Genome-wide copy number and genotype analysis of 10 cervical cancer cell lines by array CGH and SNP array showed highly complex large-scale alterations. A comparison between array CGH and SNP array revealed that the overall concordance in detection of the same areas with copy number alterations (CNA) was above 90%. The use of SNP arrays demonstrated that about 75% of LOH events would not have been found by methods which screen for copy number changes, such as array CGH, since these were LOH events without CNA. Regions frequently targeted by CNA, as determined by array CGH, such as amplification of 5p and 20q, and loss of 8p were confirmed by fluorescent *in situ *hybridization (FISH). Genome-wide, we did not find a correlation between copy-number and gene expression. At chromosome arm 5p however, 22% of the genes were significantly upregulated in cell lines with amplifications as compared to cell lines without amplifications, as measured by gene expression arrays. For 3 genes, *SKP2*, *ANKH *and *TRIO*, expression differences were confirmed by quantitative real-time PCR (qRT-PCR).

**Conclusion:**

This study showed that copy number data retrieved from either array CGH or SNP array are comparable and that the integration of genome-wide LOH, copy number and gene expression is useful for the identification of gene specific targets that could be relevant for the development and progression in cervical cancer.

## Background

The development of cervical cancer is a multi-step process. This process starts with infection of the epithelial layer with high-risk human papilloma virus (HPV). Cervical intraepithelial neoplasia (CIN) grade I, II and III, representing reversible stages of disease, is believed to develop towards neoplasm. From CIN I onwards genomic alterations and aneuploidy can be detected [1,2] and when disease is progressing towards CIN II and III, genetic alterations become more evident [3]. HPV E6 and E7 can induce cellular transformation due to interaction with cellular proteins involved in cell cycle control, apoptosis and genomic instability [4]. The induction of genomic instability seems to be particularly important for the establishment and development of an invasive tumour [5].

So far, different studies have investigated genomic alterations in cervical cancer by means of metaphase comparative genomic hybridization (mCGH) and microsatellite marker analysis for the detection of loss of heterozygosity (LOH). Chromosomal changes involving loss of 2q, 3p, 4p, 4q, 5q, 6q, 11q, 13q and 18q regions and gain of 1q, 3q, 5p and 8q regions at various stages of disease have been identified [1,6-8]. Some of these imbalances have been associated with decreased survival such as LOH of 18q [9] or with the transition from severe dysplasia to invasive carcinoma such as gain of 3q [10]. However, few genes, targeted by these genomic imbalances and possibly involved in carcinogenesis, have been discovered due to the lack of resolution when applying mCGH [11] whereas classical LOH studies are laborious. Array comparative genomic hybridization (array CGH) is an established high resolution method to study the whole genome for chromosomal amplifications and deletions [12]. Although array CGH has relatively few printed elements on the arrays, the strength of this technique is the high spatial resolution [13]. A limitation of array CGH is the lack of genotype information. Therefore, it does not provide information about regions of LOH without copy number alteration (CNA) such as mitotic recombination or gene conversion events. The under-reprepresentation of LOH in copy number methods may be very high with up to 2/3 of LOH events being undetected because of copy-neutral and copy-gain LOH events, as was found in pancreatic cancer [14]. Recently, high-density oligonucleotide-based single polymorphism arrays (SNP arrays) have been used to identify copy number and LOH of chromosomal regions [15,16]. Appropriate software tools for the detection of CNA from SNP data are becoming available. The advantage of a combined SNP-CGH approach is the identification of allele specific gain and loss by SNP array and the robust copy number detection by array CGH.

Different factors, including changes in genome copy number, can disrupt proper gene functioning. A few studies showed correlations between chromosome CNA and gene expression changes of the affected regions [17,18]. In this study, genome-wide LOH and copy number analyses of 10 cervical cancer cell lines were investigated using SNP and CGH arrays and the sensitivity to detect CNA by the 2 platforms was compared. We aimed at identifying common regions of gains and losses and determining the influence of CNA on gene expression to find genes involved in cervical carcinogenesis.

## Results

Genome-wide LOH and copy number analyses of 10 cervical cancer cell lines were investigated using SNP and CGH arrays to identify common regions of amplification. Also the two platforms were compared in their sensitivity to detect CNA. Furthermore, data from gene expression arrays were combined with array CGH data to investigate an association between gene expression and copy number.

### CGH and SNP-LOH analysis

**Figure 1:**
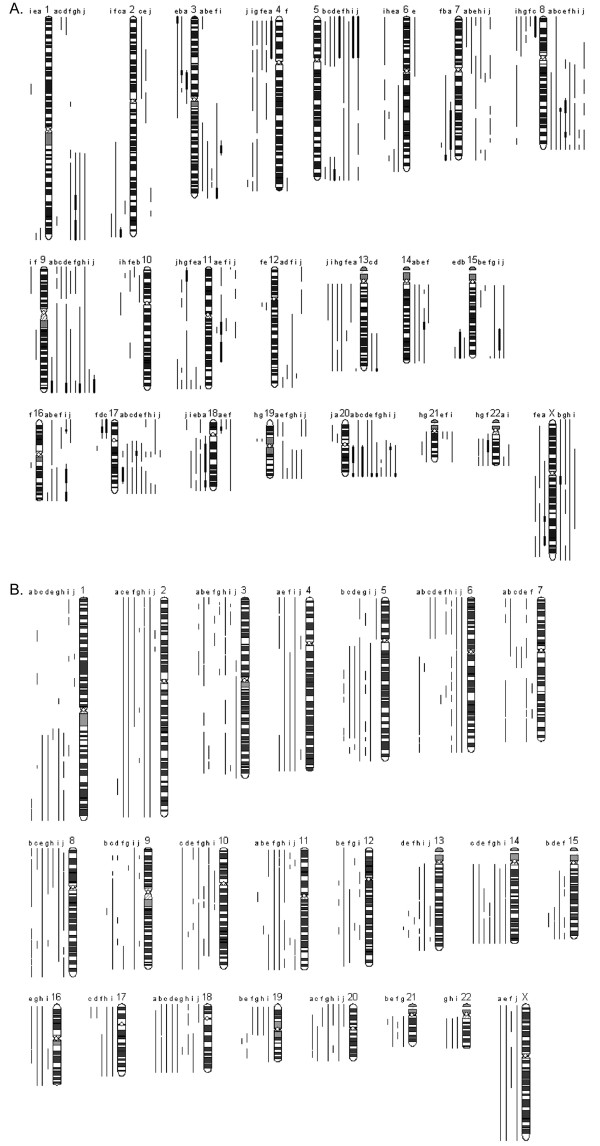
Chromosomal gains and losses in cervical cancer cell lines. A) Array CGH results are depicted by lines to the right of the chromosomes as gains and on the left as losses. Thick solid lines represent regions with amplifications of more than 1 copy loss. B) SNP-LOH results show LOH as lines on the left side of the chromosomes. The letters above the lines indicate the cell lines: a) CSCC7, b) CSCC1, c) CC11-, d) CC11+, e) CaSki, f) CC8, g) CC10b, h) CC10a, i) SiHa and j) HeLa.

Various genomic alterations affecting the majority of chromosomes were found in cervical cancer cell lines. The CGH findings for all amplifications, gains and losses detected in these cell lines are displayed in Figure 1A. Gains or amplifications of 5p, 5q, 8q, 9q, 17q and 20q occurred in most of the cell lines. Physical loss of chromosomal regions at 4p, 8p, 11q, 13q and 18q, occurred in at least half of the cell lines. Regions frequently targeted by gain or loss with small overlapping regions were present at 8p, 8q, 9p, 11q and 17q. The minimal region of overlap at 8p23 involved *TUSC3*, a tumor suppressor gene that showed loss in 5 cell lines. At 8q24, the minimal region of overlap included *c-Myc *which showed gain in 8 out of 10 cell lines. Amplification at 9q33-q34 included *endoglin*, a gene involved in tumor angiogenesis and predictive for metastasis in cervical cancer [19]. Loss at 11q25 involved 2 members of the IgLON family of cell adhesion molecules, *OPCML *and *HNT*. The minimal region of overlap at 17q11-q21 included *ERBB2 *and *TOP2A*, genes that are amplified in breast cancer [20]. As for SNP-LOH, most chromosome arms were affected by LOH in the cell lines (Figure 1B). More than 5 overlapping regions of LOH were evident for chromosome region 5q, 6, 8p, 10q, 11q, 14q, 18 and 20p.

### Comparison between SNP and BAC microarray

**Figure 2:**
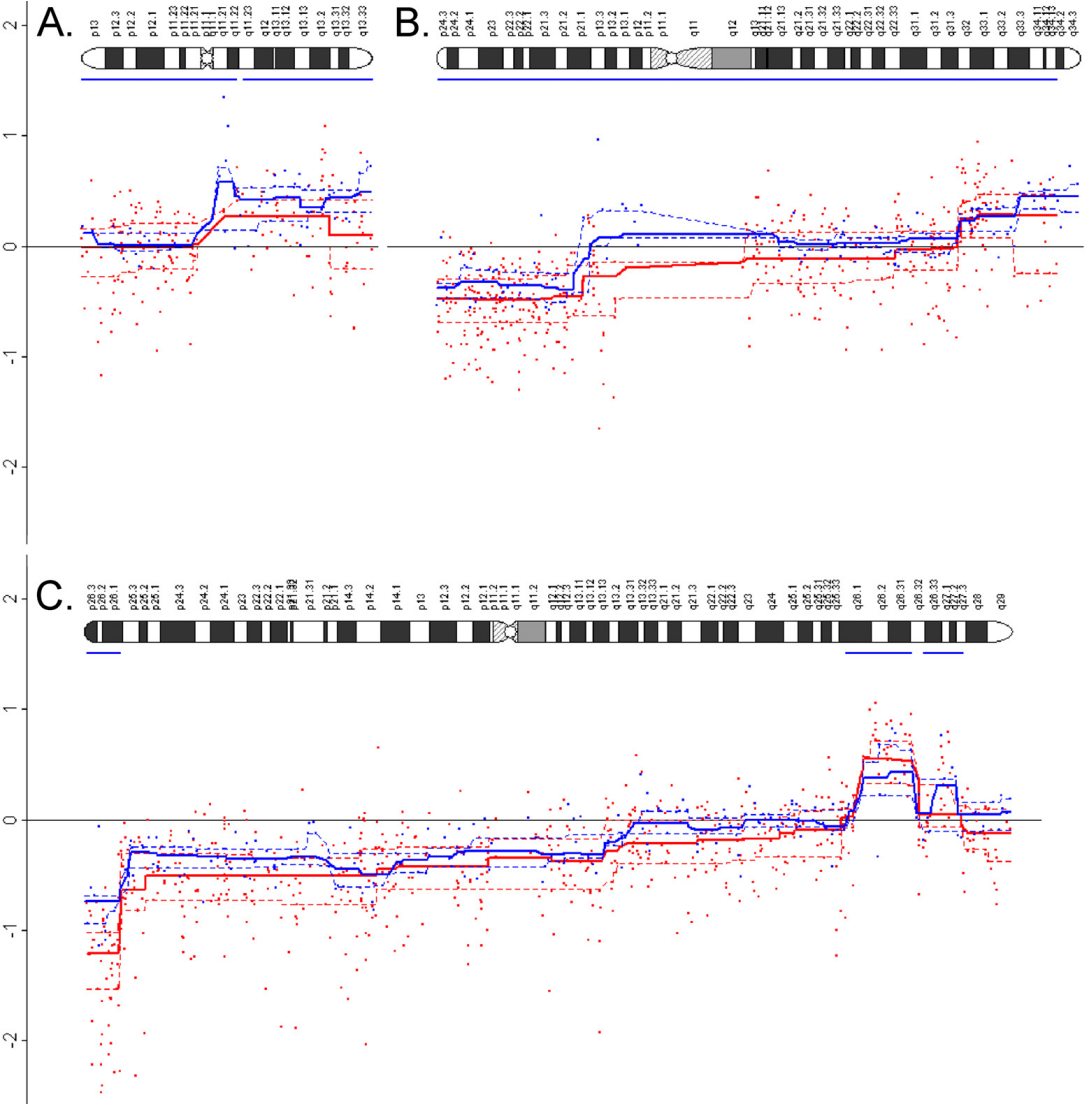
Complex genomic alterations of chromosome 20 (A) and chromosome 8 (B) of SiHa and chromosome 3 of CaSki (C). Below each chromosome ideogram, SNP array data are shown in blue and array CGH data in red. The blue line directly beneath the ideogram depicts LOH, whereas the blue line shifting around the baseline (0) shows copy number retrieved from SNP array data. The dotted blue and red lines represent the confidence intervals for the copy number of the SNP and the CGH array, respectively.

Concordance between array CGH and SNP array in the detection of CNA was investigated. Overall, the BAC and SNP arrays identified exactly the same regions of gain in 91% of cases, when taking an average of all cell lines (Table 1). Similarly, the detection of the same regions with loss between the 2 techniques was on average 94% (Table 1). Subsequently, we investigated the overlap between copy number loss and LOH and copy number gain and LOH for both platforms. For LOH to occur, measured as a reduction in copy number, physical loss is required. Thus all cases with copy number reductions are expected to have undergone LOH. In case of physical loss, around 60% was observed to encompass regions of LOH (Table 2). Overall, copy number reductions as measured by array CGH showed a higher overlap with LOH than SNP array (Table 2), namely 66% for array CGH and 57% for SNP array, when taking an average of all cell lines. The occurrence of physical gain involves a change in copy number and thus a change in the proportion of genotypes which would imply a call for LOH. Around 28% of copy number gain was found to cover regions of LOH (Table 2) and frequencies were comparable between SNP array and array CGH. Also, the overall percentage of LOH in copy neutral, copy gain and copy loss events was investigated for each cell line. The average frequency of LOH from all cell lines was 21–26% for loss, 17% for gain and 60% for copy neutral events (Table 3). Although array CGH and SNP array are comparable in the level of detection of copy number gain and loss, alterations in regions of LOH were often not found by copy number analysis because these LOH changes did not result in CNA (Figure 1 and Table 3). For example, 6 of 10 cell lines showed LOH of 6p while array CGH detected physical loss of 6p in only one cell line. This suggests that the other 5 cell lines underwent mitotic recombination upon loss of one arm, thereby not affecting copy number. Examples of complex genomic alterations are shown for chromosome 8 and 20 of SiHa and chromosome 3 of CaSki (Figure 2). In these examples (Figure 2A and 2B), LOH is caused by loss of one chromosome, accompanied by duplication of the remaining chromosome and subsequent deletions and translocations. The copy number data from SNP array as deduced from the dChip data (in blue lines) and array CGH (red lines) clearly follow the same pattern in these examples, supporting their compliance.

### Correlation between DNA copy number and gene expression

To correlate the chromosomal changes to alterations in gene expression profiles, we have searched for changes in the expression of genes located in the regions targeted by chromosomal gains and deletions. This was done by combining gene expression data from Affymetrix focus arrays with array CGH data. The average gene expression values of all cell lines were used as a reference for each cell line to determine increases or decreases in gene expression. Genome-wide, no correlation was observed between copy-number and expression. Since 5p, 8p23, 8q24, 9q33-q34, 11q25 and 20q are regions with many alterations, we focused on these areas. For 20q we compared cell lines harboring amplifications with cell lines containing gains since no cell line was devoid of 20q gain or amplification but we did not find significant differences in gene expression. Neither did we find significant changes in gene expression at 8p23, 8q24 and 11q25. At 9q33-q34, cell lines with amplification (CSCC7, CC11+, CC10A, CC10B, SiHa and HeLa) showed a significantly higher expression compared to cell lines with gains or no CNA correlation for 9 out of 86 genes (10%); *POMT1*, *FLJ35348*, *LHX3*, *NDOR*, *SH3GLB2*, *ST6GALNAC4*, *CDK9*, *PTAN1 *and *TRAF2 *(p < 0.05, T-test), of which the latter 3 have been implicated in tumorigenesis. Fourteen of 64 genes (22%) at 5p showed a significantly higher expression in cell lines with amplification (CaSki, SiHa and HeLa) compared to cell lines with gains or no CNA (Table 4).

### Validation of genetic alterations by FISH and LOH

**Figure 3:**
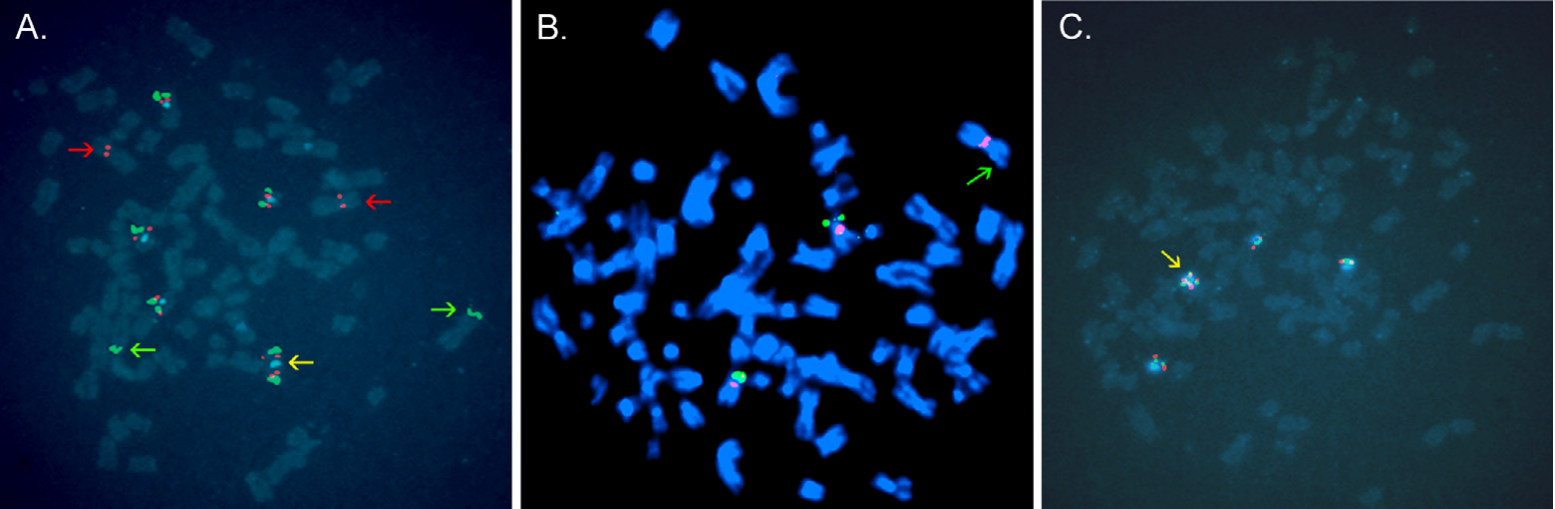
FISH of metaphase preparations depicting copy number alterations. A) CaSki – 5 chromosomes 5 (centromere 5-blue; TRIO-green; SKP2-red); one isochromosome 5p (yellow arrow), 2 translocations of RP11-1150G22, encompassing TRIO (green arrow) and 2 translocations of RP11-624K2, encompassing SKP2 (red arrow). B) CaSki – 3 chromosomes 8 (centromere 8-red, TUSC3-green) with loss of one copy the BACs encompassing TUSC3, RP11-44L18/184C1 (green arrow). C) HeLa – 4 chromosomes 20 (centromere 20-blue, ZNF-217-red, CYP24A1-green); one isochromosome 20q (yellow arrow).

**Figure 4:**
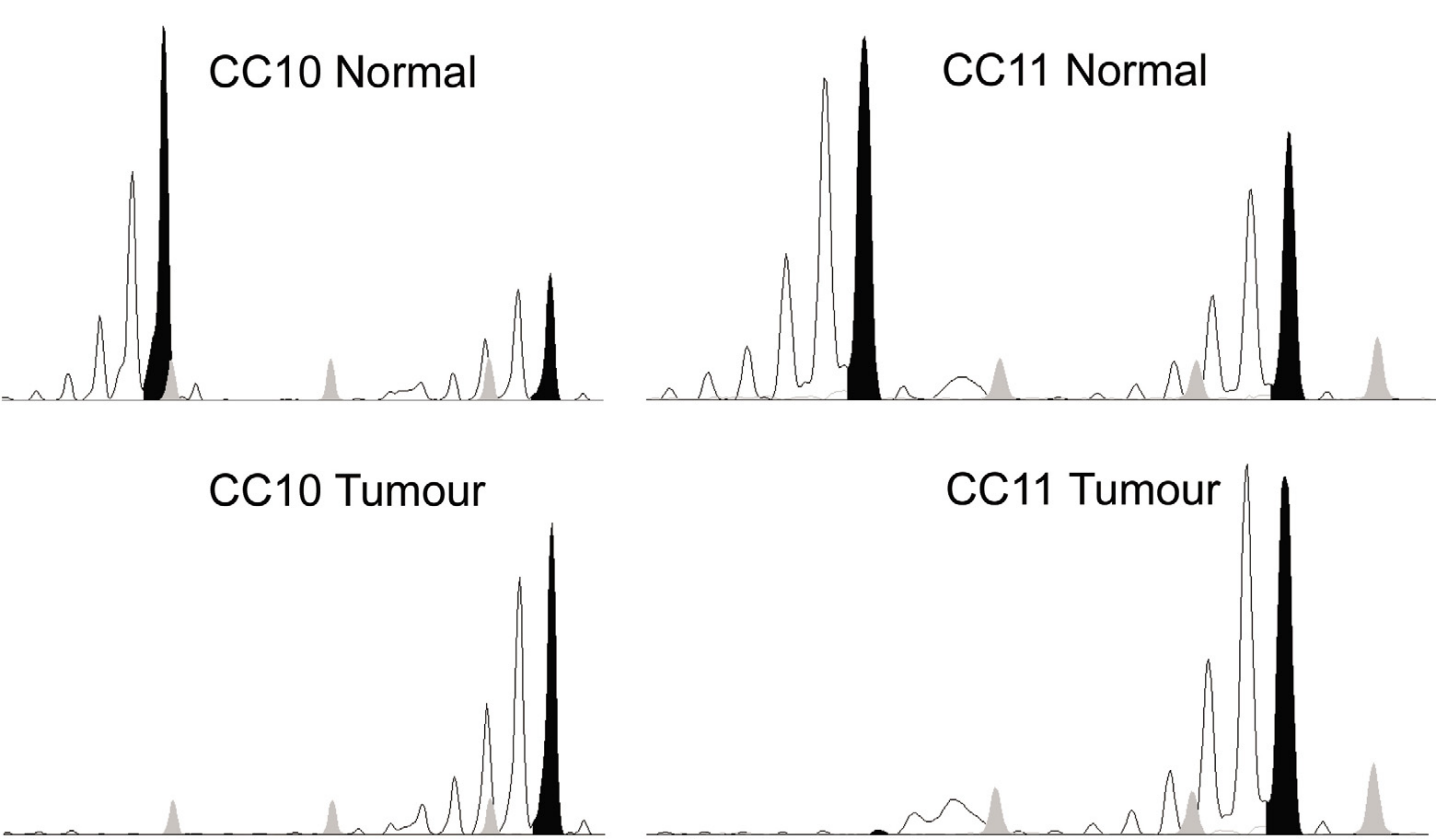
LOH at 6p21.3 is demonstrated for microsatellite marker C125. Tumour samples from which cell lines CC11 and CC10 originated were analyzed for LOH. In black the particular alleles are depicted and in grey the marker is shown.

The DNA copy number retrieved from the array CGHs was validated by FISH. Based on the gained, amplified or lost regions of 5p, 20q and 8p, BACs were selected that covered potential oncogenes or tumour suppressor genes. FISH analysis on all cell lines was performed using the BAC clones encompassing *SKP2*, *TRIO*, *hTERT*, *GDNF *and *ANKH *genes on 5p, *TUSC3 *on 8p and *ZNF-217*, *CYP24A1*, *MYBL2 *and *AIB1 *on 20q (Table 5). Besides additional copies of the area covered by BACs at 5p, other complex genetic alterations such as translocations and inversions were detected (Figure 3A). *TUSC3 *is a putative tumour suppressor gene at 8p that is covered by 2 overlapping BAC clones, RP11-44L18 and RP11-184C1 BAC. Loss of the area at 8p was concordant between array CGH and FISH for CC10A, CC11- and SiHa (Figure 3B). Signals for BACs at chromosome 20q were equal to the number of centromere signals or showed additional copies (Table 5 and Figure 3C), indicating that the whole arm or the chromosome was gained, which is in agreement with the array CGH data (Figure 1A). Overall, FISH assays confirmed the accuracy in which DNA CNA were detected by array CGH for these loci and highlighted the gains and amplifications of the *ZNF-217*, *CYP24A1*, *MYBL2 *and *AIB1 *genes at 20q, *SKP2*, *TRIO*, *hTERT*, *GDNF *and *ANKH *genes at 5p and the loss of the *TUSC3 *gene at 8p. LOH of 6p detected by SNP array in cell line CC11-, CC11+ and CC10A was confirmed by LOH analysis of the tumors from which the cell lines originate, using microsatellite markers (Figure 4).

### Validation of gene expression by qRT-PCR

**Figure 5:**
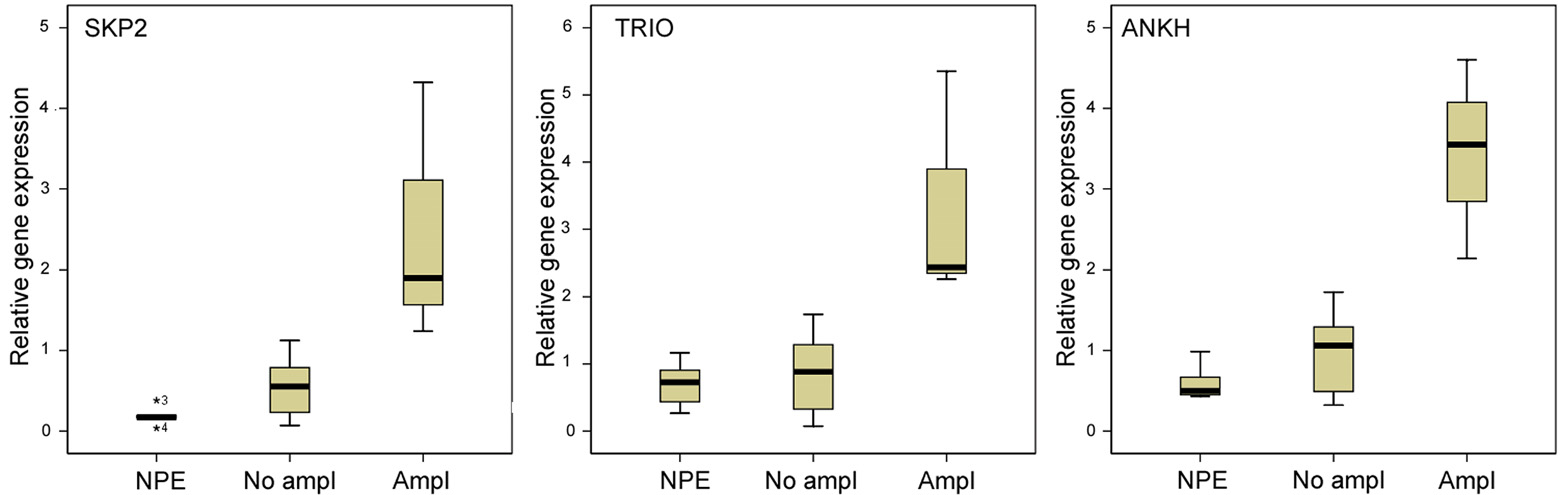
Box plots show differences in gene expression measured by qRT-PCR of SKP2 (p = 0.003, ANOVA); TRIO (p < 0.001, ANOVA) and ANKH (p < 0.001, ANOVA) in cell lines with and without amplification of 5p and normal cervical epithelial cell cultures (NPE). Ampl: amplification.

To validate the microarray data, upregulated expression of 3 potential oncogenes (*SKP2*, *ANKH *and *TRIO*) were analyzed by qRT-PCR. These genes were located on 5p that was shown to be gained or amplified by CGH in half of the cell lines. Microarray data showed upregulation of *ANKH *in 2 of the 3 cell lines with amplification of 5p which did not reach statistical significance. Five normal cervical epithelial cell cultures (NPE) were included in the analysis to investigate the difference in expression level between normal and tumour cells, with and without amplification, from the cervix. Expression was significantly different between cell lines with 5p amplification, cell lines with 5p gains or no 5p alterations, and NPE (Figure 5). At chromosome arm 8p, deletion of the tumour suppressor gene *TUSC3*, as found by FISH analysis (Table 5 and Figure 3B), was evident in CaSki, SiHa, CC10A, CC11- and CC11+. No probe set on the expression array was available for *TUSC3*. Therefore, qRT-PCR was carried out on all cell lines. Only SiHa and CaSki showed a substantial drop in expression, compared to the expression levels of the other cell lines and NPE (data not shown).

## Discussion

The present study describes genome wide LOH and copy number analyses of 10 cervical cancer cell lines using SNP and CGH arrays to identify common regions of amplification and deletion. Also, the sensitivity to detect CNA by the 2 platforms was compared. Furthermore, integrated copy number and gene expression analyses were performed to investigate an association between CNA and gene expression.

CNA retrieved from array CGH and genotype information analyzed from SNP arrays revealed very complex large-scale changes. Chromosome arms that were found to be gained and/or amplified in most of the cell lines by array CGH in this study were 5p, 8p, 8q, 9p, 9q, 17q and 20q. Especially gains of 5p, 8q and 20q have been frequently reported in cervical cancer [6,7,10,21-25]. Also the 1q and 3q arms, often found to be gained [7,10,21,24-26], were overrepresented in 5 of the 10 cell lines. Gains of 17q and chromosome 9 are less consistently reported [6,22]. Gain of 8q24, present in 8 cell lines, included *c-Myc *in the minimal region of overlap. Amplification of the *c-Myc *oncogene is a frequent event in cervical tumors, found in 25% to more than half of the tumours [27,28]. Chromosome arms affected by loss in half, or more, of the cell lines were 4p, 8p, 11q and 13q of which especially 4p and 13q have been reported regularly in cervical cancer [7,10,22,25,26,29]. Six cell lines showed loss of 11q25 that includes *OPCML *in the minimal region of overlap. *OPCML *is a tumour suppressor gene in epithelial ovarian cancer [30]. Although the genotype data have to be interpreted with some caution because of the use of cell lines, many of the frequent changes that we observed were in concordance with previous studies.

The SNP-LOH array data showed that most chromosome arms were affected by LOH in the cell lines. More than 5 overlapping regions of LOH were evident for chromosome region 5q, 6p, 6q, 8p, 10q, 11q, 14q, 18p, 18q and 20p. LOH of the majority of these chromosomes, specifically 5q, 6p, 8p, 10q, 11q and 18, has been reported previously as frequent events in cervical cancer [9,31,32]. Whereas LOH of 8p resulted in copy number loss, LOH of 6p did not result in CNA for most cases, indicative of mitotic recombination events.

A comparison between CGH and SNP array revealed that the overall concordance in detection of the same areas with CNA was on average 91% for gain and 94% for loss (Table 1). Thus both platforms are comparable in the detection of particular regions with gain or loss. Additionally, the use of high-density SNP arrays showed that cervical cancer cell lines harbor multiple regions of LOH which are not detected by methods which screen for CNA such as array CGH. Accordingly, comparison of array CGH and SNP array in genome-wide analysis shows that detection of CNA by SNP array is reliable and has the added advantage of providing genotype information. However, LOH was not consistently detected by SNP array because many copy number changes did not result in true reduction to homozygosity since most of the cervical cancer cell lines are multiploid (Figure 2C and Table 2). As expected, the areas with gain and amplification showed less LOH than regions with deletion. The overlap between copy-reduction regions and LOH was 10% higher for array CGH than for SNP array (Table 2), which suggests that array CGH is slightly better in assigning the correct regions for CNA. Although some regions of LOH are missed, thereby particularly underestimating the amount of LOH in areas with gain and amplification, an estimation was made for the distribution of LOH in regions with loss, gain and copy-neutral regions. We found that copy-neutral and copy-gain regions accounted for around 75% of all LOH identified (17% represented copy-gain LOH) while LOH associated with copy-reduction occurred at a rate of about 25% (Table 3). These percentages are similar to a recently published paper from Calhoun et al. that described only 32% of LOH events to be associated with copy-reduction [14]. Further improvement in the data from the SNP arrays may be possible using higher density SNP arrays, thereby increasing resolution and the ability to assess subtle copy number changes.

Genome-wide, we did not find a correlation between copy-number changes and expression. Lack of an association may be due to the different histological types from which the cell lines originate (Squamous carcinoma, Adenocarcinomas and Adenosquamous carcinomas) with complex genetic aberrations. Besides increased DNA dosage which may result in overexpression, transcription is influenced by other factors such as the presence and quantity of transcription and repressive factors; CpG methylation; histone methylation and acetylation status, and possibly microRNA's [33]. Thus, amplification or deletion on its own does not have to be a prerequisite for a change in gene expression. Amplification of 5p, however, correlated with a significant higher gene expression for 22% of the genes, including *SKP2*, *TRIO*, *OSMR*, *RPL37 *and *PDCD6 *(Table 4). The latter genes were previously reported in cancer, either associated with increased expression or with growth stimulatory properties [34-38].

Array CGH findings of CNA at 5p, 8p and 20q were confirmed by FISH analysis. Gains and amplifications at 5p, as found by array CGH, were validated by BACs covering candidate oncogenes, *SKP2*, *TRIO*, *ANKH*, *GDNF *and *hTERT*. *SKP2 *is important for cell cycle progression and its inhibition decreases proliferation of tumour cells [39]. Besides gain of the region covering the *SKP2 *gene, we found a significant increase in expression between normal cervical epithelial cells, tumour cells without amplification and tumour cells with amplification. Correlation between amplification of chromosome 5p and increased expression of SKP2 was previously shown in HPV-immortalized keratinocytes [40]. Upregulated expression of *SKP2*, *ANKH *and *TRIO*, as determined by qRT-PCR in cell lines with 5p amplification, confirmed gene expression data from Affymetrix focus arrays. *TRIO*, which has a putative role in cell cycle regulation, was found to be amplified and highly expressed in bladder cancer [35] that associated with proliferation and invasive phenotype. *ANKH*, which is involved in tissue calcification [41], was found to be amplified in bladder cancer [35] and small cell lung cancer cell lines [42]. Thus, upregulation of expression through amplification may be the case for *TRIO*, *ANKH *and *SKP2 *in some neoplasms, including cervical cancer, as indicated by our data. This may hold for *hTERT *and *PRKAA1 *as well since it was reported that amplification at 5p in cervical cancer correlated with increased gene and/or protein expression [43,44]. The telomeric region at 8p includes a candidate tumour suppressor gene, *TUSC3 *[45,46]. However, physical loss of this region did not correlate with a decrease in gene expression of *TUSC3*, as determined by qRT-PCR. All cell lines showed gains or amplifications of 20q, a chromosome arm which is often amplified in cancer, including cervical cancer [10,22,24,47]. At 20q increased copy numbers, detected by array CGH, were confirmed by FISH. Candidate oncogenes, *ZNF-217*, *CYP24A1*, *MYBL2 *and *AIB2*, encompassing the region analyzed by FISH, were previously reported to be amplified in other types of cancer as well [48-50]. However, no significant difference in gene expression was found between tumours with gain and tumours with amplification of 20q. Also in oesophageal adenocarcinoma, no correlation was detected between amplification of *ZNF-217*/*CYP24A1 *at 20q and increased mRNA expression [51].

## Conclusion

From this study we conclude that copy number data retrieved from either array CGH or SNP are comparable. The SNP array provided a reliable copy number as well as genotype information, which was suitable for the analysis of complex genetic alterations present in cervical cancer cell lines. Frequently occurring gains, amplifications and deletions on chromosome arms 5p, 20q and 8q were verified by FISH. Amplification at 5p associated with overexpression of candidate oncogenes. Accordingly, the possibility to integrate genome-wide LOH, copy number and gene expression is useful for the identification of gene specific targets that could be relevant for the development and progression in cervical cancer.

## Methods

### Cell lines, cultures and patient material

Human cervical cancer cell lines, HeLa, SiHa, CaSki (ATCC, Rockville, MD), CSCC1, CSCC7, CC8, CC10A, CC10B, CC11-, and CC11+ [52] were maintained in RPMI 1640 (Life Technologies, Grand Island, NY), supplemented with 10% fetal calf serum, streptomycin and penicillin at 37°C in a humid atmosphere containing 5% CO_2_. CC10A, CC10B, CC11- and CC11+ are sub clones from the same cell line, CC10 and CC11 respectively. Five normal primary cervical epithelial cell cultures (NPE) were prepared as described [53]. Two archival formalin-fixed, paraffin-embedded cervical carcinomas were obtained from the tissue bank of the Department of Pathology (LUMC, Leiden, The Netherlands).

### DNA and RNA isolation

DNA from all the cultured cell lines was isolated using the salting out procedure according to Miller and Polesky [54]. Total RNA was isolated using TRIzol reagent (Gibco BRL/Life Technologies, Breda, The Netherlands) from cells that were grown to approximately 60% confluence in 250 ml culture flasks (Greiner, Frickenhausen, Germany). Total RNA was cleaned with Rneasy cleanup system columns (Qiagen GmbH, Hilden, Germany).

### Array CGH

Array CGH was performed as previously described [55]. Slides containing ~3,500 BACs in triplicate were made at the Leiden University Medical Centre. The particular BAC set used (Welcome Trust Sanger Institute (UK)) comprises clones spaced at ~1 Mb density over the full genome, a set of subtelomeric sequences for each chromosome arm, and a few hundred probes selected for their involvement in oncogenesis. Information regarding the full set is available in the "Cytoview" window of the Sanger Center mapping database site, Ensembl[56]. The test/reference ratios were normalized for the median of the ratios of all features. The BioConductor package Limma (Smyth and Speed 2003) version 2.6.0 was used to perform background correction with method 'Edwards' and within array normalization with method 'Printtiploess', between array normalization was not performed. After normalization the replicate spots were averaged. Probes with less than 2 valid replicate values, and probes that showed a standard deviation above 0.1 between the replicate values were excluded. Chromosomal regions demonstrating a ratio of between 1.11 and 1.41 were scored as 'gained' and a ratio of 1.41 or greater was scored as 'amplified'. Chromosomal regions showing a ratio of between 0.84 and 0.73 were scored as lost whereas a second threshold for loss was set for regions showing a ratio of less than 0.73.

### SNP 10K array

Affymetrix 10K SNP array (Santa Clara, CA) containing 11,555 biallelic polymorphic sequences randomly distributed throughout the genome, excluding the Y chromosome, was used. The median distance between SNPs is ~105 kb. The average heterozygosity for these SNPs is 0.37, with an average minor allele frequency of 0.25. DNA samples, including two control DNA samples from Affymetrix, were assayed according to the manufacturer's manual protocol (GeneChip Mapping Assay manual). Genotyping was performed using GDAS (Affymetrix, Santa Clara, CA) and copy number values were calculated using dChip version 1.3. Stretches of homozygous SNPs equal or longer than 24 calls were defined as LOH.

### Gene expression microarray

Affymetrix focus arrays (Santa Clara, CA), representing 8,793 human sequences from the NCBI Refseq database, were used for mRNA expression profiling according to the manufacturer's instructions. Data files of all microarrays were normalized using the Robust Multi-Chip Average (GCRMA) method [57]. Quality control was performed on the arrays using Affy-Probe Level Model (AffyPLM) [58]. Microarrays for each cell line were performed in duplicate. The signal intensities of the replicates were averaged after normalization. To identify differentially expressed genes between cell lines with and without gain, amplification or deletion of a chromosome arm or region, unpaired two-sample t-tests were performed.

### Integrative analysis

To compare the data from the 3 high dimensional techniques; array CGH, SNP array, and expression array, the copy number and expression values were transformed to contain log 2, median centered values. Access to complete microarray data sets will be available at NCBI Gene Expression Omnibus. Normalization of array CGH data and expression arrays resulted in values that are equivalent to log2 intensity values. The data retrieved from dChip version 1.3 from the SNP arrays showed a copy number of 2 for unaffected probes on a linear scale. These data were divided by 2 and log2 transformed. A per gene median centering to zero was performed. Array CGH smoothing was performed using a smoother that retains sharp transitions between regions with genomic alterations. The correlation between gene expression and physical genomic alterations was determined by computing the Pearson correlation between the expression of each gene to the smoothed array CGH value of the nearest BAC on the chromosome. The resulting p-values were subjected to the Benjamini and Yekutieli false discovery rate procedure [59]. The data retrieved from dChip version 1.3 from the SNP arrays showed a copy number of 2 for unaffected probes on a linear scale.

### Fluorescent in situ hybridization (FISH)

Metaphase preparations of all cell lines were obtained using colcemid according to standard procedures [60]. BAC probes for the genes selected on chromosome 5, chromosome 8 and chromosome 20 (Table 5) were ordered from the BACPAC Resource Centre at Children's Hospital Oakland Research Institute (Oakland, CA). All BAC probes were fluorescein-12-dUTP- or digoxigenin-12-dUTP-labelled (Roche, Basel, Switzerland). The following centromere probes were used: the pG-A16 probe for centromere 5; the D8Z2 probe for centromere 8; and the p3.4 probe for chromosome 20. All centromere probes were kindly provided by J. Wiegant (Department of Molecular and Cellular Biology, LUMC) and labelled with biotin-16-dUTP (Roche, Basel, Switzerland). Triple-colour FISH experiments were performed as previously described [61]. Centromere and BAC signals of 20 metaphases were analyzed per probe combination per cell line.

### Microsatellite analysis

Genomic DNA was isolated from flow-sorted tumour cell subpopulations derived from formalin-fixed, paraffin-embedded cervical carcinomas as previously described [62]. Eight fluorescein-labelled primer pairs of microsatellite markers comprising MOGC, D6S265, C125, TY2A, TNFα, D6S273, D6S294 and D6S1666 [63], spanning altogether 26 MB from 6p22.1 to 6p12.1, were used. PCR, electrophoresis and analysis was performed as described previously [64]. Comparing normal and tumour DNA, a reduction of more than 50% of one allele was assigned as complete LOH.

### Quantitative real-time PCR (qRT-PCR)

qRT-PCR was performed as described previously [65]. Expression of the genes of interest was normalized by geometric averaging of multiple internal control genes (CPSF6, HNRPM, EEF1A1, RPL11 and RPL13) using the Genorm program [66]. Out of 5 normalization genes the best 3 were selected using this program; EEF1A1, RPL13 and RPL11. Primer sequences for RPL11 are 5'-ACTTCGCATCCGCAAACTCT (FWD) and 5'-AAGGTGTTGGAGCAGXTCACA (REV). Primer sequences for EEF1A1, RPL13 were reported previously [65]. Commercially available primers were used for TUSC3, ANKH and TRIO (Superarray, Frederick, USA). Primer sequences for SKP2 are: 5'-CCTATCACTCAGTCGGTGCTATGA (FWD) and 5'-AATCGTGCCAGATGGTACCCT (REV). To determine differential gene expression between normal cervical epithelial cell cultures (NPE), cell lines with and without deletion, gain or amplification, one-way ANOVA was performed.

## Authors' contributions

JK performed RNA isolation, preparation of RNA for microarray hybridization of gene expression arrays, qRT-PCR experiments, analysis of array data and preparation of the manuscript. JO performed (integrative) statistical analysis of arrays, interpretation of data and contributed to the preparation of figures. TW supervised hybridization of SNP arrays, contributed to the design of the study and critically reviewed the manuscript. KS supervised technical aspects of array CGH hybridization and critically reviewed the manuscript. JK performed array CGH hybridization. AG contributed to the design of the study and critically reviewed the manuscript. GK contributed to the design of the study and critically reviewed the manuscript. GF contributed to the design of the study and critically reviewed the manuscript. EJ performed DNA isolation, preparation of DNA and microarray hybridization of Affymetrix 10K SNP arrays, FISH experiments, analysis of array data and contributed to the preparation of the manuscript. All authors read and approved the manuscript.

## Figures and Tables

**Table 1:** Percentage of gains and losses, determined for array CGH and SNP array data.

	Gains			Losses		
Cell line	SNP (%)	CGH (%)	Con. (%)	SNP (%)	CGH (%)	Con. (%)
CSCC1	20.6	26.9	88.3	14.9	10.4	94.6
CSCC7	16.9	16.4	93.9	16.7	12.3	95.4
CC8	35.7	30.2	88.6	12.5	11.3	89.6
CC10A	10.3	23.5	86.9	19.7	8.2	83.4
CC10B	15.9	11.2	94.4	21.7	17.4	95.4
CC11-	16.4	13.9	92.9	2.4	1.8	99.3
CC11+	11.0	14.5	93.9	3.5	1.8	98.3
CasKi	20.7	30.5	87.6	22.6	13.8	91.1
SiHa	12.6	13.7	92.7	23.1	20.9	94.1
HeLa	30.3	24.0	86.2	5.4	3.9	98.2

Average	19.0	20.5	90.5	14.3	10.2	93.9

The percentage of concordance between both techniques is shown in the latter column of gains and losses. Abbreviations: Con. is concordance.

**Table 2:** Percentage of overlap between LOH and copy number gains and losses.

	% overlap between LOH and CNA			
	
	Gains		Losses	
Cell line	SNP (%)	CGH (%)	SNP (%)	CGH (%)
CSCC1	41.3	30.2	39.4	52.1
CSCC7	13.1	14.9	72.0	92.0
CC8	17.8	16.9	4.8	18.2
CC10A	26.6	15.7	73.9	82.2
CC10B	22.1	35.4	45.6	42.4
CC11-	32.3	38.9	77.3	90.1
CC11+	9.1	6.9	57.2	87.5
CasKi	60.8	52.4	52.4	45.7
SiHa	38.1	49.6	95.7	97.0
HeLa	19.5	15.8	46.6	54.8

Average	28.1	27.7	56.5	66.2

**Table 3:** Percentage of LOH determined for copy neutral, copy gain and copy loss events.

	% LOH					
	
	Copy neutral		Gain		Loss	
Cell line	SNP (%)	CGH (%)	SNP (%)	CGH (%)	SNP (%)	CGH (%)
CSCC1	25.7	30.4	43.9	41.8	30.3	27.7
CSCC7	17.5	19.8	12.8	14.1	69.6	66.0
CC8	79.1	78.5	19.0	15.3	1.7	6.2
CC10A	62.7	77.6	5.9	7.9	31.3	14.4
CC10B	52.3	59.6	12.5	14.2	35.2	26.2
CC11-	80.1	80.7	14.7	14.9	5.2	4.4
CC11+	83.6	86.1	5.5	5.5	10.9	8.4
CasKi	53.4	60.0	20.5	26.6	26.0	13.7
SiHa	54.6	54.2	8.1	11.5	34.3	37.3
HeLa	63.3	74.1	25.8	16.6	10.9	9.4

Average	57.2	62.1	16.9	16.8	25.5	21.4

**Table 4:** Genes significantly overexpressed in cell lines with amplification of 5p (T-test, p < 0.05).

Gene symbol	Chromosomal Location
ARSB	chr5p11
RPL37	chr5p13
SKP2	chr5p13
OSMR	chr5p13.1
TARS	chr5p13.2
RAD1	chr5p13.2
SUB1	chr5p13.3
RNASEN	chr5p13.3
POLS	chr5p15
SDHA	chr5p15
TRIO	chr5p15.1-p14
NDUFS6	chr5p15.33
TRIP13	chr5p15.33
PDCD6	chr5pter-p15.2

**Table 5:** Aberrations determined by FISH analysis at chromosomes 5, 8 and 20.

**Gene**	**Location**	**BAC clone**	**1**	**7**	**8**	**10a**	**10b**	**11-**	**11+**	**S**	**C**	**H**
SKP2	5p13.2	RP11-624K2	-	-	-	-	-	-	-	G	G	G
TRIO	5p15.2	RP11-1150G22	-	-	-	-	-	-	-	G	G	G
hTERT	5p15.33	RP11-117B23	-	-	-	-	-	-	-	G	G	G
GDNF	5p13.2	RP11-914H3	-	-	-	-	-	-	-	G	G	G
ANKH	5p15.2	RP11-99I1	-	G	-	G	G	G	-	G	G	G
TUSC3	8p22	44L18/184C1	-	-	-	L	-	L	L	L	L	-
ZNF-217	20q13.2	RP4-724E16	G	-	G	G	G	-	-	-	-	G
CYP24A1	20q13.2	RP5-1075G21	G	-	G	G	G	-	-	-	-	G
MYBL2	20q13.12	RP11-153L9	G	-	G	G	G	-	-	-	-	G
AIB1	20q12	RP11-1151C1	G	-	G	G	G	-	-	-	-	G

(-) no aberration; G – gain; L – loss. Cell lines: 1 – CSCC1; 7 – CSCC7; 8 – CC8; 10a – CC10A; 10b – CC10b; 11 – CC11-; 11+ – CC11+; S – SiHa, C – CaSki, H – HeLa.
